# How to sustainably build capacity in quality improvement within a healthcare organisation: a deep-dive, focused qualitative analysis

**DOI:** 10.1186/s12913-021-06598-8

**Published:** 2021-06-18

**Authors:** Peter D. Hibbert, Martin Basedow, Jeffrey Braithwaite, Louise K. Wiles, Robyn Clay-Williams, Robert Padbury

**Affiliations:** 1 Australian Institute of Health Innovation, Macquarie University, 75 Talavera Rd, Sydney, New South Wales Australia; 2 IIMPACT in Health, Allied Health and Human Performance, University of South Australia, Adelaide, South Australia Australia; 3 Department of Surgery and Perioperative Medicine, Southern Adelaide Local Health Network, Adelaide, South Australia Australia; 4 College of Medicine and Public Health, Flinders University, Adelaide, South Australia Australia

**Keywords:** Quality improvement, Quality of health care, Qualitative research, Patient safety

## Abstract

**Background:**

A key characteristic of healthcare systems that deliver high quality and cost performance in a sustainable way is a systematic approach to capacity and capability building for quality improvement. The aim of this research was to explore the factors that lead to successful implementation of a program of quality improvement projects and a capacity and capability building program that facilitates or support these.

**Methods:**

Between July 2018 and February 2020, the Southern Adelaide Local Health Network (SALHN), a network of health services in Adelaide, South Australia, conducted three capability-oriented capacity building programs that incorporated 82 longstanding individual quality improvement projects. Qualitative analysis of data collected from interviews of 19 project participants and four SALHN Improvement Faculty members and ethnographic observations of seven project team meetings were conducted.

**Results:**

We found four interacting components that lead to successful implementation of quality improvement projects and the overall program that facilitates or support these: an agreed and robust quality improvement methodology, a skilled faculty to assist improvement teams, active involvement of leadership and management, and a deep understanding that teams matter. A strong safety culture is not necessarily a pre-requisite for quality improvement gains to be made; indeed, undertaking quality improvement activities can contribute to an improved safety culture. For most project participants in the program, the time commitment for projects was significant and, at times, maintaining momentum was a challenge.

**Conclusions:**

Healthcare systems that wish to deliver high quality and cost performance in a sustainable way should consider embedding the four identified components into their quality improvement capacity and capability building strategy.

**Supplementary Information:**

The online version contains supplementary material available at 10.1186/s12913-021-06598-8.

## Background

Given the burden imposed on health systems by ageing populations, technological changes, and more recently the COVID-19 pandemic, delivering high quality healthcare in a cost-effective manner remains a challenge [1]. A key characteristic of healthcare systems that deliver high quality and cost performance in a sustainable way is a systematic approach to capacity and capability building for quality improvement [2–5].

Quality improvement (QI) can be defined as: securing understanding of the complex healthcare environment; applying a systematic approach to problem solving; designing, testing, and implementing changes using real-time measurement for improvement; and making a difference to patients by improving safety, effectiveness and experience of care [6]. QI capacity and capability building leverages the inherent self-sustaining ability of organisations and systems to recognise, analyse and improve quality issues by controlling and allocating available resources more effectively [2]. Organisations can use these resources to support the delivery of their core strategic priorities, such as improving care pathways, continuously improving, and enhancing access to services by patients [3]. If capacity and capability building in QI is indeed a pre-requisite for high functioning health systems, what are its features when undertaken sustainably and continuously over a period of years?

QI, and associated capacity and capability building, is now a frequently used method to attempt to improve health systems in high, medium and low income countries [7–9]. However, fidelity of QI methods is often variable and projects may be led by professionals who lack the expertise or resources to instigate the changes required [7]. Scientific approaches for scale-up of programs that improve healthcare systems [8, 9] have been developed, but there has been insufficient attention to sharing the lessons of successes and failures [7, 8]. Therefore, given the considerable resources spent on QI, understanding the features of sustainable QI programs that are generalisable to other organisations is important.

An atypical example of a long-term capacity and capability building program for QI is at the Southern Adelaide Local Health Network (SALHN), in South Australia, Australia. SALHN’s Department of Surgery and Perioperative Medicine has a 15-year history of implementing and sustaining QI projects via an internally developed capability and support program known locally as the Continuous Improvement Program (CIP). What is unusual, although by no means unique [5], is the length of time (15 years) that the CIP has been in place. This means that the CIP has had time to evolve and foster a continuous support infrastructure, experienced personnel, and corporate memory.

### Southern Adelaide Local Health Network (SALHN)

SALHN is a network of health services in the southern suburbs of Adelaide, South Australia. SALHN comprises a major tertiary and teaching hospital (Flinders Medical Centre), a regional community hospital (Noarlunga Hospital), mental health services, sub-acute services, and primary care clinics. SALHN manages around 700 acute hospital beds.

### The Continuous Improvement Program (CIP)

The Department of Surgery and Perioperative Medicine initially used methods derived from Intermountain Healthcare in Utah, USA, developed primarily by Dr. Brent James [10, 11]. However, over time other evidence-based methods such as Lean [12] and the Model for Improvement [13] were integrated to provide a toolkit of QI techniques. CIP also incorporated principles from SALHN’s Redesigning Care Unit which used process redesign and lean thinking to improve patient flow across the organisation [14]. SALHN have developed an eight-step process (Define the Problem, Breakdown the Problem, Set a Target/Mission Statement, Cause Analysis, Interventions, Implementation, Evaluate/Assess Impact, and Continuous Improvement – Table 1) to organise the toolkit of QI techniques and to be their overarching QI structured methodology (SALHN Continuous Improvement Framework) [15]. The Surgical Department established an Improvement Faculty to coach, mentor, and train staff, and to progressively improve their methods. Essentially, over a decade and a half, the CIP went from discrete and bolted-on *projects* to a coherent, coordinated *program* of work.

**Table 1:** Southern Adelaide Local Health Network’s 8 Step Problem Solving Process in their Continuous Improvement Framework

1	2	3	4	5	6	7	8
Planning	Diagnostic Phase			Intervention Phase		Assess Impact	Continuous Improvement
Define the Problem	Breakdown the Problem	Set a Target/ Mission Statement	Cause Analysis	Interventions	Implementation	Evaluate/Assess Impact	Continuous Improvement
Define the gap between current and expected performance. Identify stakeholders. What is the evidence there is a problem?	Thoroughly explore and understand the current state. Using data, process mapping and observation, break down and clarify the problem. What is the evidence now that there is a problem?	Using the breakdown from step 2, refine the problem statement. Set SMART targets for the improvement: • **S**pecific • **M**easureable • **A**ppropriate • **R**ealistic • **T**ime-bound	In cross functional teams, explore all possible contributing causes to the problem. Identify the root causes of the contributing causes by asking “why, why, why,…?”	Identify and assess all possible interventions for each cause. Conduct small trials. PDSA (Plan-Do-Study-Act) cycles	Develop an implementation plan in consultation with affected areas. Implement the plan in a controlled manner. Ensure appropriate controls are in place to support staff in the affected areas.	Monitor the results to assess the effectiveness of the interventions. If ineffective, understand why and adjust the interventions as appropriate.	Once proven effective, standardise for the new process. Ensure the process is monitored so it can be continuously improved. Consider where else the learnings from this could be applied (other wards, units, campuses, etc.)

References: Steps of Problem solving. St Vincent’s Hospital Melbourne. S Craig/C Cummins 2018

SA Health. Improving Care Methodology: Supporting Practice and Process Redesign

CIP was initially implemented in surgical contexts at SALHN in 2004. SALHN executive then supported the expansion of CIP in 2018 from the Surgical Department into a SALHN-wide program, across all services.

The aims of SALHN CIP are at organisation level – to build capability as a high performing health service and develop a collaborative continuous improvement culture; and at a participant level - to learn a standard approach to continuous improvement and problem solving which is applicable across all levels of staff, to help the organisation create reliable systems and provide safe, high quality care. CIP is designed to provide an opportunity for participants to learn how to identify and solve problems in the workplace they are passionate about, partner to work within a team, and use improvement methodology to step through the problem systematically.

The CIP’s education component commences with introductory 3½ day off-site training sessions with presentations by both senior staff from SALHN and members of the Improvement Faculty. The initial topics covered include an overview of the CIP’s history, an outline of its key objectives, the evolution of QI and the need for a systematic improvement framework. Days 2 and 3 involve small group work, with an introduction to the diagnostic tools used in the CIP, such as breaking down the problem, process mapping, brainstorming, multi-voting and Pareto charts, organised by the SALHN Continuous Improvement Framework [15, 16]. The importance of measurement and the significance of human factors science to QI are stressed in Improvement Faculty presentations. Presentations on Day 4 include standardisation in clinical practice and the significance of reducing, where possible and desirable, unnecessary variation in healthcare. The sessions also provide opportunities for groups to work through practical cases using CIP methodologies.

After the introductory 3½ day off-site training sessions, participants then select QI projects to work on and lead. The QI projects are based on improving clinical problems e.g., related to patient safety, length of stay, or patient experience – see Additional file 1: Details of CIP projects for examples. The participants invite clinicians (doctors, nurses, allied health of all levels of expertise) and administrative staff who are likely to understand the reasons why a clinical problem exists, onto a QI project team to collectively address the problem. They may be identified within or across clinical departments depending on the scope and type of clinical problem or pathway that they are attempting to improve. More than one participant from the CIP may be on a project team.

Some 3 months after the introductory training sessions, the CIP participants attend a follow-up, full-day symposium, where each team presents on the progress of their project. The symposium is relatively informal and interactive, with an emphasis on allowing participants to learn from the experience of others.

After a further 3 months, participants in the CIP graduate. In this final one-day session, participants need to have demonstrated that they have used CIP methods to initiate a service improvement. The project does not need to be completed; however, significant progress must be presented. Minimum requirements are set for graduating participants, including the identification of a problem worth solving and evidence to justify the project, brainstorming the contributing factors to the problem, creating a cause and effect diagram, identification of outcome measures and how they are to be used, and the application of a run chart.

Between July 2018 and February 2020, SALHN conducted three Continuous Improvement Programs (CIP1, CIP2, CIP3) covering 82 individual projects. Additional file 1 provides details of all projects. A summary of participant numbers in the three Programs is shown below in Additional file 1.

### The Improvement Faculty

The SALHN’s Improvement Faculty comprised six members at the commencement of the CIP1. Faculty members were initially nurses from the Department of Surgery and Perioperative Medicine who had many years of experience in undertaking QI projects. From CIP1 and ongoing, the Faculty traini and mentor other clinicians from other departments in SALHN who participate in CIP to join the Faculty. SALHN’s Improvement Faculty and associated researchers were interested in understanding the principles and components of the CIP and the improvement projects themselves that were generalisable to other organisations and programs attempting to undertake sustainable QI in healthcare. Too little research focuses on this meso-level capacity – mostly, studies are of a specific project (infection control or use of radiology in the clinical microsystem [17, 18]) or system wide studies [19–21]. The aim of this research was to explore the facilitators and barriers to implementing the CIP QI projects and the influence of the scaled CIP capacity and capability building program on the success of these QI projects (Table 2).

**Table 2:** Study definitions

**Capacity:** refers to having the right number and level of people who are actively engaged and able to conduct improvement [22].	
**Capability**: people having the confidence, knowledge and skills to lead the improvement [22].	
**Implementation:** put in place interventions that can result in true improvements in quality [23].	
**Lean:** A process-improvement methodology articulated as Lean Thinking principles which were developed in the context of elaborately transformed goods such as motor cars. Lean healthcare is the application of “lean” ideas in healthcare facilities to minimize waste in every process, procedure, and task through an ongoing system of improvement [12, 14].	
**Model for Improvement:** The Model for Improvement is made up of a set of fundamental questions that drive all improvement and the Plan-Do-Study-Act (PDSA) Cycle. Combined, the three questions (What are we trying to accomplish?; How will we know that a change is an improvement?; What changes can we make that will result in improvement?) and the PDSA Cycle are the framework called the Model for Improvement [13].	
**Plan Do Study Act (PDSA):** PDSA is a quality improvement method utilizing a framework for an efficient trial-and-learning methodology. The basic concept of which is that an improvement intervention follows four phases: 1. Planning what to do 2. Doing the improvement 3. Studying its impact 4. Acting on the results of that study to revise and improve on what was done. Multiple PDSA cycles are often needed to make successful changes [13].	
**Quality Improvement (QI):** securing understanding of the complex healthcare environment; applying a systematic approach to problem solving; designing, testing, and implementing changes using real-time measurement for improvement; and making a difference to patients by improving safety, effectiveness, and experience of care [6].	
**Sustainability:** Sustainability should be viewed as a characteristic of healthcare which must run through and moderates other domains. Healthcare should be considered not only in terms of what can be delivered to an individual today, but also to the population in general and the patients of the future [24].	

## Methods

### Study methods

By way of executing a deep-dive study of the CIP, interviews of CIP project participants and the Improvement Faculty and observations of project meetings were conducted. Qualitative thematic analysis of data collected from these sources was conducted. The consolidated criteria for reporting qualitative research (COREQ) checklist is shown in Additional file 2.

### Data collection and participants

#### Interviews

Staff who participated in CIP and the Improvement Faculty were interviewed for the research. The researchers briefly presented to all three introductory CIP sessions to let participants know that the research was being undertaken and that they may be contacted with a request for interview. The researchers purposively selected 20 CIP participants from a range of departments across SALHN to capture a range of experience and health services professions. The researchers anticipated reaching saturation at around 12–15 interviews, however, as it was felt important for a range of departments to be represented, 20 CIP participants were therefore invited. Eight participants were selected from both CIP1 and CIP2 and four from CIP3 and invited to be interviewed.

There were twenty-three participants (*n* = 16, 70% females) who agreed to participate in the study comprising 19 single interviews (CIP1 (*n* = 8), CIP2 (*n* = 8) and CIP3 (*n* = 3)) and one group interview of four Improvement Faculty members. Of the 19 clinicians, there were ten nurses, seven doctors, one pharmacist, and one physiotherapist. Interviews were conducted between September and November 2019 by PH and MB, who are male and have extensive experience in qualitative research (see Additional File 2, COREQ questions 2 and 5 for more details). The researchers had no formal pre-existing relationship with study participants (see Additional File 2, COREQ questions 6–8 for more details). Participants were approached to participate by email. Interviews were conducted onsite at SALHN, and there was no-one else present.

The aim of the interviews was to identify, from this experienced, hands-on cohort, the barriers and facilitators to implementing QI projects that have been supported by a sustained and scaled capacity and capability support program and identify how the CIP facilitated the success of the projects. Interviews were semi-structured and comprised approximately 25–30 questions depending on responses (see Additional file 3 for the schedule of interview questions). Each single interview took between 30 and 45 min, whilst the group interview took around 60 min. Following the earlier CIP1 and 2 interviews, questions with CIP 3 participants were slightly modified to further explore issues that had emerged. All interviews were recorded with permission and transcribed.

#### Observation of project teams

To gain a deeper understanding of the mechanics, interpersonal interactions, experiences, norms of the teams, and barriers and facilitators to projects, researchers (MB and PH) attended team meetings of individual projects. Researchers PH and MB attended seven meetings of four CIP project teams. Project team members could be CIP participants or not – details are shown in Additional file 4. The number of attendees at each meeting ranged from 5 to 10 (mean 8), with nearly two-thirds (*n* = 36/56, 54%) of attendees being female. The mean meeting duration was around 70 min (range 0.5 to 3 h).

During the observation, the researchers took an ethnographic approach, observing how work was done, including the behaviours, interactions, and communication between the team members [25]. In addition, to guide the process, a template was developed from a meeting observation guide originally produced by NHS England [26]. The template included sections on QI principles, team behaviour, and meetings standards (see Additional file 5). Notes were also taken during observations and written up as soon as possible to avoid recall bias.

Given the sensitive nature of the research, it was made clear to the project teams that all information would be treated confidentially and dealt with in a considerate manner. Project teams were also made aware that findings were to be published in ways so as to not identify individual participants.

### Data analysis

Interview transcripts and notes from observations were inductively and thematically analysed independently and iteratively by two researchers, who then compared themes. Braun and Clarke’s Framework for thematic analysis was used as the guiding framework [27]. nVivo v.12 (QSR International Pty. Ltd.) was used as the analysis tool. The interviews and the observations of the team meetings were analysed using the same set of codes developed in nVivo. Project team observations were also deductively analysed separately using the categories from the NHS England template [26]. Data saturation was reached. Supporting quotes for each theme were extracted from the transcripts and presented with the results.

### Ethics

Requisite ethics and governance approvals were obtained before research commenced – SALHN Human Research and Ethics Committee: HREC/18/SAC/369 and Office for Research: SSA/19/SAC/162.

## Results

There were four themes identified from the analysis of factors that lead to successful historical implementation of CIP projects and the overall Program that facilitates or support these: an agreed and robust QI system, a skilled faculty to assist improvement teams, active involvement of leadership and management, and an understanding that teams matter. These are explored in turn, together with another theme from the interviews, the relationship between safety culture and QI, which is then followed by barriers to successful improvement projects. Shorter quotes are displayed in italics in text and longer quotes are presented in Table 3.

**Table 3:** Illustrative quotes from interviews with CIP participants

Quote no.	Quotes
**Theme: An agreed and robust quality improvement methodology**	
1	“I have to say it worked; we came out with some good outcomes and a project that was in small, biteable chunks that we could then actually deliver some change and then the learnings that we took from that, I presented to different divisions and it sort of stimulated a lot of work throughout the network and got a lot of attention.” (Interview no.1, doctor)
2	“The CIP project taught me to identify the problem rather than just start with the solution. So you’re not just jumping straight into finding answers. What’s the data actually telling us now?” (Interview no.9, doctor)
3	(in relation to brainstorming sessions …) “Seeing people getting engaged – that was quite rewarding and especially when they picked things up that I thought would work” (Interview no.1, doctor).
4	“It was quality, it was enjoyable, it was good education and, I think again, just to get people together across the network and hospital working on a similar methodology and learning, I think’s really important” (Interview no.5, doctor).
5	“There’s no doubt that there’s quite a few things that need attention and this kind of project can improve the quality of care. Now I – when I do my clinical work, I see a lot of areas that could use a continuous improvement project” (Interview no.12, doctor).
6	“It’s not just me doing it for a nursing cohort, but it’s more so to the benefit of the patient and the outcomes going to benefit the service” (Interview no.15, nurse).
7	“I think it’s a different way of looking at things and I think it’s a good way and I think we can use it in our environment” (Interview no2, doctor).
8	“… another tool in the toolbox…and the more tools you’ve got, the more options you have” (Interview no.7, nurse).
**A skilled faculty to assist teams and other skills to support projects**	
9	“Having the facilitator was great. Just having someone go through processes, for example, with the Pareto chart, you get one graph and you think, I need to go another round of voting. And just having someone there … being able to soundboard … who’s done it before. Having a coach was fantastic.” (Interview no.16, nurse)
10	“I think we also were given a lot of ongoing support which made the project and information more relevant, so I could check in and ask questions if I needed to” (Interview no.7, nurse).
11	One interviewee valued the ability “to soundboard from someone who’s done it before, and being given a little bit of guidance, I think, was very helpful for me” (Interview no.16, nurse).
12	“I think it would be helpful in the long-run if you could have assistance in data collection and things like that, because it’s difficult to do that when you’re also doing your job” (Interview no.4, nurse).
13	(referring to previous CIP graduates …) “So they bring a greater knowledge and research base to projects that you engage in” (Interview no.6, nurse).
**Active involvement of leadership and management**	
14	“It showed good leadership right from the start, and that people at the higher levels were committed, which I think was good” (Interview no.8, nurse).
15	“If the person right at the top of our organisation is engaging in the CIP project and allowing people in those executive roles to participate, then I think that’s leading by example” (Interview no.7, nurse).
16	“It’s given some priority and some importance. And there’s been investment of time and effort to run the Program” (Interview no.14, doctor).
17	“So I think that sends an important message that they support it and they think it’s worthwhile and that they were prepared to implement this across the network” (Interview no.5, doctor).
18	“I think that executive probably need to be a bit more cognisant of the time this takes and being given the accessibility to be able to perform the process” (Interview no.6, nurse).
19	“I can say that he (Director) was very supportive of the project, like exceptionally supportive, without his help it wouldn’t have gone ahead” (Interview no.9, doctor).
**An understanding that teams matter**	
20	“The various perspectives from different discipline and different areas (which) helped shape our project” (Interview no.14, doctor).
21	“Having that multi-disciplinary atmosphere, where there’s people from all levels of clinical, managerial side of things, we probably talked a lot more and met with people that we wouldn’t have otherwise talked to at all. So we got a lot more perspectives on things. That was really good” (Interview no.3, nurse).
22	“Seeing people getting engaged – that was quite rewarding and especially when they picked things up that I thought would work” (Interview no.1, doctor).
23	“I enjoy the fact that the nurses got a sense of achievement when they saw that the work they put in actually brought them some improvement to a problem that they had been struggling with for a very, very long time” (Interview no.7, nurse).
24	“I enjoyed hearing other people’s problems and how they solved them. That was probably the most enjoyable part of it and it was interesting seeing the process of other people going through the same thing and the challenges that they faced and they overcome them” (Interview no.10, pharmacist).
25	“You need to get the right mix of people in the room and to have the right network, and to think ‘outside the square’ as to who’s actually going to be valuable in the room” (Interview no.1. doctor).
26	“We tried to have a mix of nurses/medical staff or junior doctors/senior doctors, even GPs because they want to know different things and they all came with their own spin on things” (Interview no.1, doctor).
27	“You need people that are invested in and interested in getting the outcome that you’re trying to achieve” (Interview no.8, nurse).
28	“The key facilitator is access to the right people who have the tacit knowledge” (Interview no.7, nurse).
**The relationship between safety culture and quality improvement**	
29	“Everybody was engaged in the process, and I thought – actually this is how you facilitate or implement change; you get the people in the room, you get their buy-in, they were motivated, engaged, they were really proud of themselves like, oh, we thought of something like we can do this. And that’s when I was like, yeah, I can see it” (Interview no.1, doctor).
30	“It’s making it part of a culture in a way of thinking, rather than just a process and education tool that you did once and you’re never going to use again” (Interview no.6, nurse).
31	“But once I started, I realised that’s how people come together, to help to do this kind of project. So, I thought, that’s a very supportive culture that we have here” (Interview no.12, doctor).
32	“How can they improve things is also enormously beneficial from a personal development perspective for them and it leaves people with their batteries a little bit recharged and feeling a bit better about the world, rather than sort of being rundown” (Interview no.7, nurse).
33	“So I wanted to increase my understanding of how to actually lead an improvement project. Probably one of the goals was to kind of increase my confidence in actually kind of doing a project of that scale” (Interview no.4, nurse).
34	“It strengthened my other aspects like teamwork and respecting each other, getting help from other as well” (Interview no.12, doctor)
**Barriers to successful implementation at project level**	
35	(There were) “some things that I had planned to do, but just haven’t got around to doing. I mean, there is pressure put on us for our clinical duties. That has to be a huge barrier” (Interview no.13, doctor)
36	“Everyone is exhausted by change, nobody wants to face another something new and something different and like they’re just tired of it” (Interview no.9, doctor).

### An agreed and robust quality improvement methodology

Interviewees appreciated the structured approach to the QI process, which they found beneficial to problem solving and decision-making. They valued being given a step-by-step approach to problem solving (using the structure of the SALHN Continuous Improvement Framework [15]), particularly in a healthcare system that is characteristically complex and high-pressured (Table 2, Quote 1). Many interviewees particularly valued the principles of establishing the root causes of a problem rather than the rush to find a solution (Quote 2).

A number of interviewees expressed an appreciation of the CIP’s strong process orientated methodology. One doctor expressed her surprise at the value of this approach, which was so alien to her grounding in scientific methods, such as the use of randomised controlled trials. She found the whole concept of looking at interventions in the Plan Do Study Act (PDSA) cycle and the use of different kinds of outcome measures very challenging. Several interviewees mentioned the brainstorming sessions (Quote 3). One interviewee enjoyed her exposure to the PDSA cycle, “*learning about that and how I can apply that to my everyday practice*” (Interview no.3, nurse), whilst another, who was initially sceptical, said that she enjoyed the voting process. Another expressed a similar view, saying that they approached the training with some “*anxiety and trepidation*” (Interview no.12, doctor).

The applicability of the methodology to a variety of situations (that extended outside of the QI environment) was perhaps an unexpected benefit of the training to some participants. Related to this, was the notion of having an agreed methodology, which results in people having a common understanding and language (Quote 4).

Several interviewees mentioned being attracted to the underlying robust methodology due to the positive impact that undertaking the CIP would have ultimately have on patient outcomes (Quotes 5 and 6). Some expressed the view that they would recommend the CIP because of the different but valuable skills that it brought to patient care and to their own skill set (Quotes 7 and 8).

The theme of having an agreed and robust QI system was again noted in the observations of the meetings. In all instances, the project teams had clear evidence of what they hoped to achieve from their improvement project with clearly defined objectives and timeframes. All projects were focussed on resolving problems that had a significant impact on their health service.

During observations, project teams demonstrated the use of robust diagnostic tools, using established tools such as process mapping, cause and effect analysis and Pareto charts. An observation of a team leader respectfully asking a staff member to avoid rushing to solutions was noted. Extensive use of other data was also noted, including customer surveys and complaint reports, and the determination of patient demand and clinician time through the interrogation of patient administrative and coding systems.

Teams were observed spending considerable time reviewing and discussing data, a process viewed as fundamental to their QI activities. Data were often presented in handouts provided in advance of the meetings. Examples of data reviewed in meetings included weekly patient demand, yearly comparisons of patient activity, listing of patient delays in treatment, and a time- and-motion study on tasks undertaken by clinicians. Whiteboards were frequently used in meetings, particularly during process mapping and voting exercises. One team participant, a long-term staff member, strongly supported the process by stating words to the effect of “*we have tried things in the past, but never evaluated it. The difference this time is the review of data*”.

There was much discussion about how to “read” the data and interpret it. The managers who led the meetings stressed that data should be viewed as a tool for improving the delivery of healthcare, not for performance management purposes. Furthermore, it was highlighted that people should acknowledge data around capacity and patient demand could be confronting and that care should be taken not to focus on individual performance, but instead consider the system.

### A skilled faculty to assist improvement teams

Nearly all of those interviewed considered that the Improvement Faculty, which provided training, mentorship and ongoing support to project teams, were critical facilitators to their success. Many expressed gratitude at the willingness of the Faculty to help solve problems as they arose, giving guidance to project teams on QI methodology and offering practical advice when issues were encountered (Quotes 9–10). A pre-requisite to engaging project teams was that the training needed to be high quality and enjoyable (Quote 4).

Other skill sets mentioned were having a clinical lead who was “*well informed about the process and the project and the methodology*” (Interview no.16, nurse) and “*a clinical lead – an improvement coach – I think is a must*” (Interview no.8, nurse) and support with data collection (Quotes 11 and 12). Others mentioned the need for sufficient time to tackle the problem, funding requirements, and training material and resources.

Several interviewees felt that the Improvement Faculty’s job was assisted by people who had previously undertaken the CIP and had a greater understanding of QI and its practical application. The fact that more and more people across SALHN were now “*talking the CIP language*” was seen as a significant development (Quote 13).

### Active involvement of leadership and management

Almost all interviewees recognised the importance of the supportive role played by senior management, which included both hospital executive and heads of department. Their role was deemed to be a positive influence, although the nature of their support differed. For example, the Chief Executive Officer at SALHN was noted to be a key driver of the Program, both in directing the health network’s strategy to encompass it, and in her active participation in training sessions (Quote 14). The CEO’s “*leading from the top*” was a commonly expressed view of her support for the Program and her commitment to have the whole organisation trained in the QI methodology and influencing other executives was crucial for buy-in from QI teams (Quote 15). Other senior executives were also seen as being supportive of the CIP by undertaking the training and involvement in projects as sponsors (Quote 16). There was also significant utility in the fact that senior management prioritised the CIP as an important initiative (Quotes 16 and 17).

It was commented that senior management were in a unique position to see the bigger picture of activities within their health network, and because of this, were able to assist with projects that were of significance to the organisation. Conversely, feeding up information from the “grassroots” level gave senior management insight into problems of which they may have otherwise been unaware. On the other hand, one interviewee highlighted that senior management perhaps underestimated the time commitment of staff undertaking the CIP and allowances need to be made for this (Quote 18).

Many heads of Department demonstrated their support for the CIP in practical ways, for example, by allowing time off for their staff to attend training sessions and team meetings. In some instances, they also participated in the CIP themselves with some taking carriage of particular projects. One senior medical clinician expressed the view that without the support of her Departmental Director, the project in which she was involved would not have proceeded (Quote 19).

The influence of the senior leaders was observed in one of the project meetings. The meeting leader referred to CIP and stated that “*this approach is going to be the norm*” indicating that senior staff were strongly and sustainably supporting the approach.

### An understanding that teams matter

The experience of working in a team to solve a problem was considered one of the most enjoyable aspects of the CIP by virtually all interviewees. A number of people mentioned the camaraderie that developed amongst team members, with many also highlighting the benefit of forming relationships with staff that normally they would not encounter in the workplace. Of particular benefit were the diverse perspectives that different disciplines brought (Quotes 20 and 21). The fact that tangible outcomes were achieved and shared between team members reinforced the value of the projects and contributed to a collective sense of achievement (Quotes 22 and 23). One interviewee commented on the revelation that different professions shared the same challenges (Quote 24).

During “brainstorming” sessions, it was observed that some participants initially showed a tendency to consider the issues being discussed solely from their personal perspective. As the nature of the problem being discussed emerged, it appeared that participants became more willing to consider the views of others. An example of this related to a discussion about waiting lists, with medical staff saying that they better appreciated the stressful impact that waiting lists also had on administrative staff.

Obtaining the right mix of skills in the team was also seen as crucial in conducting a QI project (Quote 25). One interviewee mentioned the benefit that could be gained from sourcing expertise external to the organisation, such as general practitioners who co-manage patients (Quote 26). A senior doctor stressed the importance of selecting the right people to attend the first brainstorming session, an action that she considered could “*make or break*” the project.

The attitude required within the team was to have an *“open-minded”* approach to problem solving and a willingness to openly discuss options and possible solutions were seen as key facilitators to a successful QI project (Quotes 27 and 28). Another interviewee referred to the benefit of having “*sensible, reflective conversations*” about what happens in your workplace (Interview no.7, nurse).

In the meetings observed of all four teams, there was clear evidence of mutual respect amongst participants. In all instances, it was noted that leaders facilitated meetings that were characterized by openness and an encouragement for “*brutal honesty*” as one leader described it. Although it was apparent in most meetings that participant involvement in proceedings was not uniformly equal, contributions were, nonetheless, sought from all those in attendance.

The important role of the leader in setting the tone for the meeting was observed. At one meeting, for example, the leader stressed the importance of having “*trust in the process*” and gave clear guidance throughout proceedings, frequently clarifying issues. It was also noted that he intentionally voted last on the contributing factors to the problem being investigated, so as not to influence others. On another occasion, in a different project group, one of the participants frequently expressed potential solutions throughout discussions as issues arose. Respectfully, she was reminded of the need to not “*jump ahead*”.

The benefits of undertaking the CIP, in terms of personal development, were raised by a couple of interviewees. One senior clinician stated that he encourages participation in the CIP “*because it’s good for your own personal growth and at the end of the day, you’re happy that you have done something for the hospital and for your patient and to society*” (Interview no.12, doctor).

### The relationship between quality improvement and culture

Some interviewees presented the view that the methodology and the project acted as a conduit to cultural change, rather than a good safety culture being a pre-requisite for QI. They felt that this way of thinking may then become the culture and a way of working (Quotes 29–31). Using the methodology and improving care can be a re-invigorating experience for staff, again providing positive cultural impacts (Quote 32). One senior nurse described the benefits she had gained in terms of her self-assurance in tackling a large and complicated problem (Quote 33). Another expressed how the CIP had developed his skills at working in a team, which again contributes to a positive culture (Quote 34).

### Barriers to successful implementation at project level

The high-level barriers to success at the QI project level included gaining support from senior and clinical staff, change fatigue, professional rivalries, the difficulty of tackling large and complex problems, and contractual issues with other organisations. However, the overwhelming barrier to the successful implementation of the CIP was time and logistics. Virtually all interviewees commented on the challenge of maintaining busy clinical and administrative workloads and undertaking the CIP (Quote 35). Some participants considered “change fatigue” a barrier (Quote 36).

Connected with the time factor were logistical challenges such as arranging meetings for what often involved a disparate group of professional and administrative staff. Senior medical staff, it was noted, were generally considered the most difficult group to get to meetings. One interviewee voiced his frustration at this aspect of his project, citing the six-week delay in getting a medical consultant and registrar together for a meeting.

## Discussion

Qualitative research, conducted via the interview and observational data collected in this study, can provide insights into the way in which QI capacity and capability activities are organised institutionally and what components clinicians and executives believe are the most important factors for their success [28]. Our study of the SALHN CIP revealed a number of factors that lead to successful implementation of clinical practice improvement projects and the overall program that facilitates or support these. At the core these were an agreed and robust QI system, a skilled faculty to assist improvement teams, active involvement of leadership and management, and an understanding that teams matter. We also found that the relationship between a strong safety culture is not necessarily a pre-requisite for QI gains to be made; indeed, they appear to be reciprocal, and our results show how QI can contribute to a strong safety culture. For most participants in the CIP, the time commitment for projects was significant and, at times, maintaining momentum was a challenge. The task of combining busy clinical and administrative workloads, whilst simultaneously undertaking time consuming projects, was invariably difficult, as were identifiable logistical challenges, such as arranging meetings.

The CIP has been sustained for 15 years at the level of the Department of Surgery and Perioperative Medicine and was then scaled up across the whole of health service in 2018. At the level of the Department of Surgery and Perioperative Medicine, sustainability was promoted by clear ownership of the Program, having been initially adapted from a number of frameworks [10–14] to suit local need [29, 30]. The Program was strongly supported by the Head of Surgery who provided steer and momentum, had a dedicated resource of the Improvement Faculty to support the Program, and was integrated into core business and priorities [29]. Benefits of undertaking the CIP training and projects were clear to those who participated, in terms of improved teamwork, more effective multi-disciplinary relationships and improvement in the delivery of quality care to patients. These characteristics were also important for successful scale up from the Department of Surgery and Perioperative Medicine to the whole of SALHN. Additionally, the CIP had key features of interventions likely to be scaled up. The Program was based on locally generated evidence of programmatic effectiveness and feasibility [29]. What had been demonstrated over 15 years at the Department of Surgery and Perioperative Medicine was that the CIP was a “scalable unit” [9] that could be adopted more widely across SALHN. CIP was associated with respected and credible senior people within the organisation [29, 30], its impacts were observable to participants [29, 30], and the CIP was highly relevant to contemporary healthcare practice and organisation [29, 30]. CIP was compatible or congruent with SALHN’s values and norms because it was developed internally [30]. The leadership of SALHN strongly aligned the outcomes of CIP with their own priorities of running a large health service in an era of rising demand and an ageing population. Their strong advocacy of the program was a key factor in its successful adoption across the health service [31]. Their willingness to invest in the Improvement Faculty as a key strategic and on-the-ground resource and the Faculty’s willingness to broaden its membership by mentoring others was also key.

**Fig. 1:**
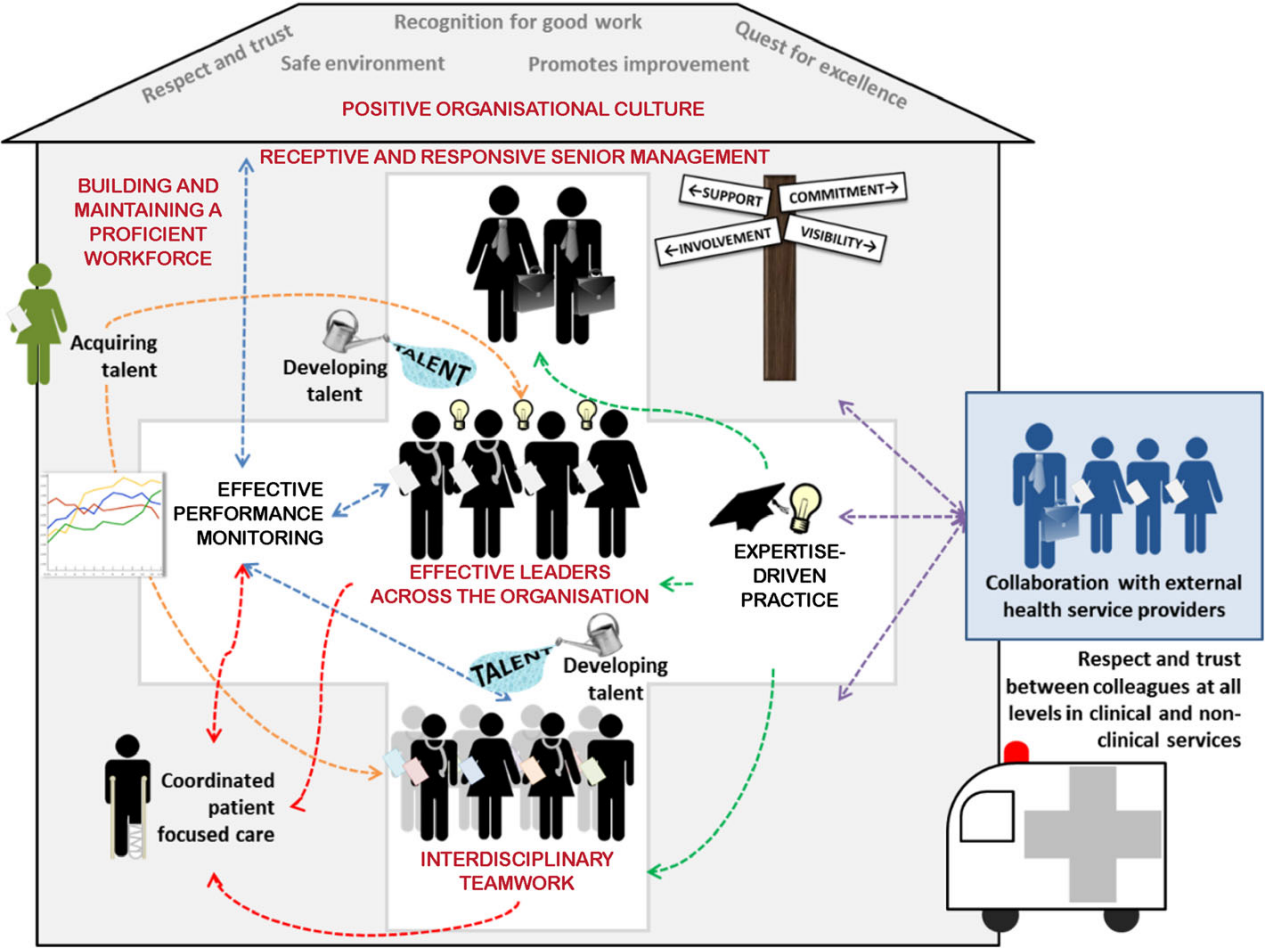
Components of high-performing hospitals [32] and ingredients necessary for a successful quality improvement program highlighted in red ovals [Adapted by permission from Springer Nature Customer Service Centre GmbH: Springer Nature, BMC Health Services Research. (High performing hospitals: a qualitative systematic review of associated factors and practical strategies for improvement. Taylor N, Clay-Williams R, Hogden E, Braithwaite J, Groene O.) COPYRIGHT 2015]

Our findings align well with our earlier systematic review on the features of high performing hospitals, i.e., those which consistently attain excellence across multiple measures of performance, and multiple departments (Fig. 1, [32]). Five of the seven characteristics of high performing hospitals were receptive and responsive senior management, effective leaders across the organisation, positive organisational culture, interdisciplinary teamwork, and building and maintaining a proficient workforce – these all featured to some degree in the CIP (Fig. 1, red font). The systematic review found that receptive and responsive senior management and effective leaders across the organisation involved deep interactions with staff, a hands-on style, and a proactive and continuous participation with improvement activities [32]. In our study, senior management, who supported staff to enhance performance were active and highly visible, were easy to speak to and actively made themselves available to interact and to jointly solve problems with staff. The relative ease of access to higher levels of management has been a further benefit of the CIP, facilitating a more efficient pathway to problem resolution. Under the auspices of the CIP, staff have found value in these improved communication channels, obviating the bureaucratic processes that are characteristic of so many large institutions. Conversely, feeding up information from the grassroots level has given senior management insight into problems to which they were previously unaware.

Qualitative systematic review evidence suggests respect and trust were key characteristics of a positive culture within high performing hospitals, manifesting in staff feeling safe to speak out, take risks and suggest ideas for improvement [32]. Interdisciplinary teamwork was characterised by strong coordination amongst disciplines and departments working together over time to achieve common goals [32]. In our study, the capacity to work in a team was a fundamental component of the various QI projects undertaken in the CIP. Having the right mix of skills and people in the room was important, particularly for the first meeting where the “tone” was set. Most participants enjoyed the experience of team activities, valuing both the camaraderie that developed and the benefits of forming relationships with staff that normally they would not engage with in an in-depth way in the workplace.

In 2019, the UK’s Health Foundation reviewed the characteristics of high performing National Health Service Trusts in England [3]. These Trusts were rated as outstanding by the regulator, the Care Quality Commission (CQC). Most of these have implemented an organisation-wide QI program, which the CQC noted in its 2017 State of Care report [33]. The CQC also emphasised that organisations with a ‘mature QI approach’ have prioritised improvement at the level of their board, developed and implemented a plan for building improvement skills at all levels of the organisation, and developed structures to oversee QI work and ensure it is aligned with the organisation’s strategic objectives [34]. The work that SALHN has undertaken with its CIP mirrors these approaches with a strategy of buy-in, visible support from executive members, and creating a faculty to support QI teams. Persisting for 15 years seems to be a further ingredient for building capacity at SALHN with the highly regarded Jönköping Country in Sweden reporting similar longevity [5].

The onsite CIP Faculty are critical facilitators to the success of the Program. The support of the Faculty through the provision of training, practical advice and mentorship to project teams has been an important element in maintaining the momentum of work being undertaken. In other studies of QI programs, it has been reported that having dedicated project leadership teams was by far the most commonly reported facilitating factor for the implementation of QI projects [35].

The relationship between safety culture and performance in healthcare is never likely to be simple and is far more likely to be complex and contingent [36]. Some of the QI project team members interviewed expressed the view that undertaking a QI project is a significant contributor to changing culture in the clinical micro-system. Working as a team with a common goal that is recognised and agreed to be a problem, with staff who do not normally closely engage with each other in a structured manner, enabled hierarchies to be flattened, and having staff generate and own the problems and solutions contributed to the perception of culture change – and the likelihood of it being realised. It is possible that a catalyst for culture change was a collective declaration by team members that there is a problem that effects the quality of care provided to patients or staff well-being that is worthy of solving [37]; that they admit that systems within their control may be contributing to the problem; and they were willing to use discretionary effort to work towards solving it. The process orientated tools may allow all opinions in the room to contribute more-or-less equally, encouraging authority gradients to flatten, and for causes of problems to be generated by clinical staff who work in the area [38]. This means that they are less inclined to feel as if the solution has been imposed upon them [39]. The strong overt support of the Program from SALHN executives and managers was likely to enhance the culture change generated from QI activities [40]. The alignment of both local safety culture at the level of QI teams and leadership at the level of the organisation is one of the three principles of the 20 year QI journey at Jönköping County in Sweden whereby improvement is best construed as both bottom-up and top-down [41].

As to weaknesses, the study was a focused, deep-dive into one health system, and may not be generalisable. A strength was to use two complementary data sources – staff interviews and observations. The observations provided validation to the interview responses and themes that were iteratively developed. The interviews were broadly representative across the three CIPs and professions, including medical staff.

## Conclusion

Our study revealed interacting components that were deemed necessary for a successful QI program. These include an active involvement of leadership and management, a skilled faculty to assist teams, an agreed and robust QI system, and an understanding that teams matter. Other healthcare systems may wish to consider embedding these components into their quality improvement capability and capacity building strategy. The time commitment for staff to undertake projects was significant which can impact on maintaining momentum. These findings align well with the contemporary literature on capability and capacity building of QI knowledge and skills.

## Supplementary Information

**Additional file 1.** Details of CIP projects.

**Additional file 2.** Consolidated criteria for reporting qualitative research checklist (COREQ).

**Additional file 3.** Interview questions.

**Additional file 4.** Details of project teams, meetings and attendees.

**Additional file 5.** Template for observations of CIP meetings.

## Data Availability

The interview questions are outlined in Additional file 3; the template for observations are shown in Additional file 5. The interview transcripts are not available due to ethics requirements and potential to identify participants.

## Abbreviations

CIP: Continuous Improvement Program
CQC: Care Quality Commission
PDSA: Plan Do Study Act
QI: Quality Improvement
SALHN: Southern Adelaide Local Health Network
