# Supplementary material for: How to sustainably build capacity in quality improvement within a healthcare organisation: a deep-dive, focused qualitative analysis
Source: BMC Health Serv Res. 2021 Jun 18;21:588. doi: 10.1186/s12913-021-06598-8 (PMC8212075; doi:10.1186/s12913-021-06598-8)
Supplement: Supplementary file 1 — Additional file 1. Details of CIP projects. [file 12913_2021_6598_MOESM1_ESM.pdf]

Additional File : Details of CIP projects

Table A1: CIP1 projects

Department/Division	Project Title
Nursing & Midwifery	1:1 Specials
Finance & Corporate Services	All Patients, Visitors, Staff have adequate car-parking availability within
Division of Medicine, Cardiac & Critical Care Serv	An unplanned admission to ICCU initiates vital treatment and stabilises the
SAPOM	Breast Category 1 Overdues
Division of Medicine, Cardiac & Critical Care	Clinical Improvement Project Ambulant Low Risk Chest Pain Pathway
Palliative Care/RAP	Community Palliative Care
Mental Health	Completion and review of risk assessment documentation in Morier Ward
Allied Health	Ensure Allied Health add value at the right time with the least waste (and
Rehabilitation, Aged & Palliative Care	Hand Hygiene compliance in Allied Health Rehabilitation Teams
Corporate Services	Hand Hygiene Products at Point of Care
TMOU, Cardiology	Heart Failure / TMOU Clinical Improvement Project
Division of Medicine, Cardiac & Critical Care Serv	Improving consumer engagement – for unplanned ICCU admission
Workforce WHS/IM Service	Improving Injury Reporting
Office of CEO	In 6 months the CEO will complete at least half a day a week of front line staff engagement time that include all sites, services and staff groups
Respiratory	Incidental Lung Nodules
Mental Health	Integrated Consumer Assessment & Rapid Engagement Project
Corporate & Support Services	Management of Un-Categorised Referrals
Mental Health	Mental Health Act Compliance
Mental Health Services	Minimum Standards for Handover - Prisoner Movement Order
Woman's & Children's	Paediatric Discharge Project

Department/Division	Project Title
Division of Medicine Cardiac and Critical Care	Patient transfers from ED to Division of Medicine Bed Process
W&C/Obstetrics & Gynaecology	Reducing postpartum haemorrhage
Mental Health Services	Standardising the Process for SALHN Adult Mental Health Community
Clinical Governance Unit	The feedback & compliments from consumers not fed back or utilised - [illegible]hs
Anaesthesia	The Surgical Safety Checklist
SAPOM	Timely access to image intensifier services in theatre at Flinders Medical
RAP	Transition Care Program - Improving Occupancy Rates and Time to
Workforce Services	Vacancy Management

Table A2: CIP2 projects

Department/Division	Project Title
RAP	Therapy time in Inpatient Rehab
Workforce	Aboriginal employment in SALHN
EDAH	AH capacity and demand
MCC	Assessing Frailty in general medical patients
MH	Community Reform - Consumer Allocation
MCC	Continuous Nursing Observation in the Emergency Department
EDMS	ED Discharge Summary Completion
Workforce	Employee Recognition Women’s and Children’s Division
SAPOM	GasTrostomy Tube Management in Endoscopy
MCC	Improving clinician engagement during MET calls in Medical wards
MCC	Improving Hospital Length of Stay in Mechanically Ventilated Patients
Neonatal Unit	Improving hypothermia rates of high risk infants in the FMC Neonatal Unit
SAPOM	Improving timeframes around men receiving results following a prostate biopsy within the SALHN Urology unit.
MH	ITO compliance
Corporate	Management of Inpatient Complaints
MCC	Medication Omissions in the ICCU
WCD	NNU staffing & work flows
EDON	Nursing and Midwifery Personal /Carers Leave
SAPOM	Outreach Aboriginal Ophthalmology Patient Management Flinders Eye Centre
WCD	Paediatric Outpatient Clinic Attendance
MCC	Patient coming from ED to ward that don’t match the handover
Workforce	Performance Review and Development
RAP	Problems in GEM Length of stay
Corporate	Recycling rates at Noarlunga Hospital falling below the contracted target of 50%
CEO Office	SALHN Imprest Management
Corporate	SALHN: Activity Based Budgeting Project
EDAH	Up to date PPGP
MCC	White blood cell count+F18

Table A3: CIP3 projects

Department	Title of Presentation
Cancer Services	The CHAMP Project
Medical Oncology	Geriatric Assessment in Medical Oncology Out Patient Clinics
Division of Medicine	TNT: Trim Needle Times
Medicine, Cardiac & Critical Care	Bedside Handover
Medicine, TMO Unit & Corporate Nursing	Intern Night Shift Debriefing Program, Growing Resilience and Wellbeing in the Intern cohort
Infection Control, CNMER, SAPOM	Hand Hygiene
SAPOM & Consumer Engagement	"My appointment was at 10!" Reducing the wait in Neurosurgery clinic
SAPOM	Four Weeks From Oesophagogastric Cancer Diagnosis tU tReatment WEEKS
SAPOM	Why was my surgery cancelled?
Workforce Services, Human Resources	Overpayments
Social Work & Counselling	Early Identification of Complex Care Needs
Allied Health	C - Closing the O - Occupational Therapy & P - Physiotherapy S - Service Gap in General Medicine
RAP	Frailty Screening
Rehabilitation & Aged Care	SWOT Social Welfare on Tobruk
RAP	A complicated admission pathway from ED to MSS, Whittaker
Executive Office	Communication with Consumers
SAPOM	DaySE's Day Surgery Emergencies
W&C Division	MAW – TIME Minimising Antenatal Wait Time
Mental Health	Assisting MH Clinicians to record and Review Physical Health Interventions

Table A4: Number of CIP Participants by course and profession type

Program	Dates	Total Participants	Medical	Nursing	Allied Health	Administration
CIP1	30/07/18 to 22/02/19	50	14	14	4	18

CIP2	26/02/19 to 26/07/19	44	11	16	6	11
CIP3	30/07/19 to 7/02/20	41	13	13	6	9
