# Supplementary material for: How to sustainably build capacity in quality improvement within a healthcare organisation: a deep-dive, focused qualitative analysis
Source: BMC Health Serv Res. 2021 Jun 18;21:588. doi: 10.1186/s12913-021-06598-8 (PMC8212075; doi:10.1186/s12913-021-06598-8)
Supplement: Supplementary file 2 — Additional file 2. Consolidated criteria for reporting qualitative research checklist (COREQ). [file 12913_2021_6598_MOESM2_ESM.pdf]

Additional File 2: Consolidated criteria for reporting qualitative research checklist

No. Item	Guide questions/description	Reported in section or page no.^
**Domain 1: Research team and reflexivity**		
Personal Characteristics		
1. Interviewer/facilitator	Which author/s conducted the interview or focus group?	Methods: Study methods, Data collection and participants, Interviews, paragraph 1.
2. Credentials	What were the researcher's credentials? E.g. PhD, MD	Methods: Study methods, Data collection and participants, Interviews, paragraph 1.<br>Additional information:<br>PH – B.App.Sc., Grad.Dip.Comp., Grad.Dip.Econ, PhD;<br>MB – B.Ec., M.P.H., PhD
3. Occupation	What was their occupation at the time of the study?	Methods: Study methods, Data collection and participants, Interviews, paragraph 1.
4. Gender	Was the researcher male or female?	Methods: Study methods, Data collection and participants, Interviews, paragraph 1.
5. Experience and training	What experience or training did the researcher have?	Methods: Study methods, Data collection and participants, Interviews, paragraph 1.<br><br>Additional information below:<br><br>PH has undertaken semi-structured interviews and focus groups with healthcare professionals, managers and executives mainly in relation to patient safety and quality improvement over the last 15 years for the purposes of research and evaluation. He is the author of 15 peer-reviewed papers which undertook qualitative analysis of patient safety incident reports.<br><br>MB has carried out qualitative research in the course of undertaking his PhD, in addition to various consultancy work. He is also

No. Item	Guide questions/description	Reported in section or page no.^
		the lead author in 5 peer -reviewed papers in which qualitative analysis is the methodology.
Relationship with participants		
6. Relationship established	Was a relationship established prior to study commencement?	Methods: Study methods, Data collection and participants, Interviews, paragraph 1.<br><br>Additional information: The researchers partially attended CIP1, 2 and 3, 3 month full day symposiums, and graduations. The researcher met some of the participants at these meetings.
7. Participant knowledge of the interviewer	What did the participants know about the researcher? e.g. personal goals, reasons for doing the research	Yes, at the CIP meetings, PH briefly presented the reasons why the research was being undertaken and its goals.<br>The participant information form that was provided with the consent form signed by the participant was explicit in its description of the purpose of the research.
8. Interviewer characteristics	What characteristics were reported about the interviewer/facilitator? e.g. Bias, assumptions, reasons and interests in the research topic	At the CIP meetings, the research was framed as a partnership between the Southern Adelaide Local Health Network and the research team.
Domain 2: study design		
Theoretical framework		
9. Methodological orientation and Theory	What methodological orientation was stated to underpin the study? e.g. grounded theory, discourse analysis, ethnography, phenomenology, content analysis	Methods: Study methods, Data analysis, paragraph 1.
Participant selection		
10. Sampling	How were participants selected? e.g. purposive, convenience, consecutive, snowball	Methods: study methods, data collection and participants, interviews, paragraph 1.

No. Item	Guide questions/description	Reported in section or page no.^
11. Method of approach	How were participants approached? e.g. face-to-face, telephone, mail, email	Methods: study methods, data collection and participants, interviews, paragraph 1.
12. Sample size	How many participants were in the study?	Interviews – Methods: study methods, data collection and participants, interviews, paragraph 1.<br><br>Observations - Methods: study methods, data collection and participants, observation of project team processes paragraph 1.
13. Non-participation	How many people refused to participate or dropped out? Reasons?	N/A
14. Setting of data collection	Where was the data collected? e.g. home, clinic, workplace	Methods: study methods, data collection and participants, interviews, paragraph 1.
15. Presence of non-participants	Was anyone else present besides the participants and researchers?	Methods: study methods, data collection and participants, interviews, paragraph 1.
16. Description of sample	What are the important characteristics of the sample? e.g. demographic data, date	Interviews – Methods: study methods, data collection and participants, interviews, paragraph 1.<br>Observations - Methods: study methods, data collection and participants, observation of project team processes paragraph 1.
Data collection		
17. Interview guide	Were questions, prompts, guides provided by the authors? Was it pilot tested?	Interviews: Additional File 3<br>Observations: Observations - Methods: study methods, data collection and participants, observation of project team processes paragraph 2.
18. Repeat interviews	Were repeat interviews carried out? If yes, how many?	N/A
19. Audio/visual recording	Did the research use audio or visual recording to collect the data?	Interviews – Methods: study methods, data collection and participants, interviews, paragraph 2.<br>Observations - Methods: study methods, data collection and participants, observation of project team processes paragraph 2.
20. Field notes	Were field notes made during and/or after the interview or	Observations - Methods: study methods, data collection and

No. Item	Guide questions/description	Reported in section or page no.^
	focus group?	participants, observation of project team processes, paragraph 2.
21. Duration	What was the duration of the interviews or focus group?	Methods: study methods, data collection and participants, interviews, paragraph 2.
22. Data saturation	Was data saturation discussed?	Methods: study methods, data collection and participants, interviews, paragraph 1.
23. Transcripts returned	Were transcripts returned to participants for comment and/or correction?	No
Domain 3: analysis and findings		
Data analysis		
24. Number of data coders	How many data coders coded the data?	Methods: Study methods, Data analysis, paragraph 1.
25. Description of the coding tree	Did authors provide a description of the coding tree?	Not applicable: The data analysis was inductive and therefore did not use a pre-existing deductive coding tree.
26. Derivation of themes	Were themes identified in advance or derived from the data?	Methods: Study methods, Data analysis, paragraph 1.
27. Software	What software, if applicable, was used to manage the data?	Methods: Study methods, Data analysis, paragraph 1.
28. Participant checking	Did participants provide feedback on the findings?	No
Reporting		
29. Quotations presented	Were participant quotations presented to illustrate the themes/findings? Was each quotation identified? e.g. participant number	Table 1
30. Data and findings consistent	Was there consistency between the data presented and the findings?	Yes. The four themes were developed from the data and are presented in sequence in the Results (Results, An agreed and robust quality improvement methodology; Results, A skilled faculty to assist improvement teams; Results, Active involvement of leadership and

No. Item	Guide questions/description	Reported in section or page no.^
		management; Results, A belief that teams matter; with associated quotes in-text and in Table 1; These results are then considered in the Discussion.
31. Clarity of major themes	Were major themes clearly presented in the findings?	Results, Paragraph 1 (and subsequent headings).
32. Clarity of minor themes	Is there a description of diverse cases or discussion of minor themes?	Results, Paragraph 1.
