# Supplementary material for: How to sustainably build capacity in quality improvement within a healthcare organisation: a deep-dive, focused qualitative analysis
Source: BMC Health Serv Res. 2021 Jun 18;21:588. doi: 10.1186/s12913-021-06598-8 (PMC8212075; doi:10.1186/s12913-021-06598-8)
Supplement: Supplementary file 3 — Additional file 3. Interview questions. [file 12913_2021_6598_MOESM3_ESM.pdf]

Additional File 3: Interview questions

Continuous Improvement Program 1 and 2

1. What CIP project are you involved in?
2. Has the Program knowledge and skills been relevant to your job and your problem to solve?
3. What other support or resources do you need to apply the knowledge and skills in the project? Do you have examples where these have been provided?
4. What is the role of senior management in relation to improvement projects? Have you seen examples of these roles being done?
5. Are you experiencing any barriers to a successful improvement project being undertaken? How have they hindered your work?
6. What do you think are the key requirements to a successful improvement project being undertaken? What, if any, have been the key requirements in your project? How has this helped your work?
7. What has been the reception or attitude to the improvement projects from clinicians who did not undertake the CIP?
8. Has anyone else in your workplace completed the training? If so, have you noticed changes in their knowledge, skills or performance? Tell me about what you have seen?
9. Prior to attending the Program what did you anticipate would be useful for your work? Now that you are doing the Program, was it more or less useful as you expected? In what ways?
10. What were your own development goals when you went into the Program? Did you achieve them? How do you know you have achieved them?
11. What are/did you enjoy most about the program? What are/did you enjoy least about the program?
12. Would you recommend the program to your peers? Why?
13. How could the Program be improved? (Hint: Program structure and contents, facilitation, materials, and the learning environment be improved?)
14. What are the benefits of the program (think individual, team, network)
15. Do you have anything further to add?

Continuous Improvement Program 3

1. Have the program knowledge and skills been relevant to your job and the problem you are trying to solve?
2. Did you have any real difficulties defining the problem?
3. Was there disagreement amongst the team? If so, how were the problems resolved?
4. What other support or resources do you need to apply the knowledge and skills in the project? Do you have examples where these have been provided?
5. What do you think are the key requirements to a successful improvement project being undertaken? What, if any, have been the key requirements in your project? How has this helped your work?
6. What is the role of senior management in relation to improvement projects? Have you seen examples of these roles being carried out?
7. Have you experienced any barriers to successfully undertaking your improvement project? How have they hindered your work?
8. Have you encountered any problems obtaining evidence to justify the existence of a problem (consider data sources, people etc.)
9. In arriving at interventions/solutions, have you encountered any problems? If so, how were they resolved?
10. How can you sustain your improvement?
11. What have you learnt from CIP and how can you share these learnings?
12. Prior to attending the program, what did you anticipate would be useful for your work? Now that you are doing the program, has it been more or less useful than you expected? In what ways?
13. What have you enjoyed most about the program? What have you enjoyed least about the program?
14. Would you recommend the program to your peers? Why?
15. How could the program be improved? (Hint: program structure and contents, facilitation, materials, and the learning environment etc.)
16. Do you have anything further to add?
