# Supplementary material for: How to sustainably build capacity in quality improvement within a healthcare organisation: a deep-dive, focused qualitative analysis
Source: BMC Health Serv Res. 2021 Jun 18;21:588. doi: 10.1186/s12913-021-06598-8 (PMC8212075; doi:10.1186/s12913-021-06598-8)
Supplement: Supplementary file 4 — Additional file 4. Details of project teams, meetings and attendees. [file 12913_2021_6598_MOESM4_ESM.pdf]

Additional File 4: Details of project teams, meetings, and attendees

Table D1: Details of project teams, meetings, and attendees

Date	Project	No. of attendees	Attendee profession	CIP3 participants	Male/Female	Meeting length	Main CIP activity
23/08/19	Team 1	7	Medical (consultant) - 1; Nursing - 3; Administrative - 2; SQ/patient advocate - 1	2	0/7	1 hour	Process mapping
26/08/19	Team 4	8	Allied Health - 8	2	4/4	0.5 hour	Pareto R2 voting
11/09/19	Team 4	10	Allied Health - 10	2	5/5	0.5 hour	Process mapping, data review
13/09/19	Team 1	7	Medical (consultant) - 1; Nursing - 3; Administrative - 2; CIP Faculty - 1	2	0/7	1 hour	Identifying problem causes
20/09/19	Team 3	5	Medical (consultant) - 1; Nursing - 2;	2	1/4	1.5 hours	Identifying problem causes

Date	Project	No. of attendees	Attendee profession	CIP3 participants	Male/Female	Meeting length	Main CIP activity
			Administrative - 1; Allied Health -1				
17/10/19	Team 2	10	Medical (consultants)-3; Medical (registrar) - 1; Medical (intern) - 4; Nursing - 1; Administrative -1	3	4/6	3 hours	Identifying problem causes, voting
11/11/19	Team 4	9	Allied Health - 9	2	6/3	0.5 hour	Data review, PDSA
**Total**		**56**			**20/36**		

Table D2 below details the impact of the problem being addressed, showing that all projects, amongst other things, attempted to improve patient outcomes.

Table D2: Teams observed and problem impact.

Team no.	Problem impact
1.	Poor patient care, including reduced satisfaction and need by staff to manage patient frustrations.

2.	Mental health effect on doctors and compromised patient safety and care
3.	Inferior patient health outcomes
4.	Increased risk of patient harm, higher length of stay, employee workload stress
