# Supplementary material for: How to sustainably build capacity in quality improvement within a healthcare organisation: a deep-dive, focused qualitative analysis
Source: BMC Health Serv Res. 2021 Jun 18;21:588. doi: 10.1186/s12913-021-06598-8 (PMC8212075; doi:10.1186/s12913-021-06598-8)
Supplement: Supplementary file 5 — Additional file 5. Template for observations of CIP meetings. [file 12913_2021_6598_MOESM5_ESM.pdf]

Additional file 5: Template for observations of CIP meetings

	SCORE IF APPLICABLE (YES/NO OR 1 – 5)[1]	COMMENTS AND OBSERVATIONS
**CIP PRINCIPLES**		
AIMS Statement that is Specific, Measurable, Applicable, Realistic and Timely		
A problem worth solving – high impact, volume, cost, evidence-based care		
Robust diagnostics – evidence of tools used		
Patient/customer feedback		
Interventions linked to causes		
PDSA cycles used		

Measurement tools used		
Reception /attitude to CIP from clinicians who did not undertake program		
	**SCORE IF APPLICABLE (YES/NO OR 1 – 5)[1]**	**COMMENTS AND OBSERVATIONS**
Senior management role in relation to CIP – supportive?		
Barriers to implementation?		
Role of Faculty and interaction with group		
**<u>BEHAVIOUR</u>**		
Mutual respect		

Honest views expressed and solicited		
Clarity of contributions		
Thoughtful debate and sound judgments made		
	**SCORE IF APPLICABLE (YES/NO OR 1 – 5)[1]**	**COMMENTS AND OBSERVATIONS**
Constructive challenge		
Innovative thinking in debate		
Constructive criticism well received		
Moving beyond 'silo-ed' member contributions		

Effective, efficient and timely management of the agenda		
Enthusiasm and pace		
Overall behaviour		
	**SCORE IF APPLICABLE (YES/NO OR 1 – 5)[1]**	**COMMENTS AND OBSERVATIONS**
<u>**MEETING STANDARDS**</u>		
On time start / finish		
Formal agenda, minutes		
Members committed		

Members participation		
Discussions focussed		
Action items		

1. Scoring Key -

* Poor. No evidence. Considerable work to be done.

** Below what should be expected. Needs attention.
